# Preferences and satisfaction of food allergy sufferers using Internet resources

**DOI:** 10.1186/2045-7022-3-S3-P126

**Published:** 2013-07-25

**Authors:** A Arens, S Schnadt, F Feidert, R Mösges, P Schmalz, N Rösch

**Affiliations:** 1 CR SANTEC, Public Research Centre Henri Tudor, Luxembourg, Luxembourg; 2 German Allergy and Asthma Association, Möchnengladbach, Germany; 3 ORL-Eich, Centre Hospitalier de Luxembourg, Luxembourg, Luxembourg; 4 IMSIE, University Hospital of Cologne, Cologne, Germany

## Background

People suffering from food allergies must study food ingredients prior to consumption. While the Internet facilitates this information acquisition, little is known about consumer experiences. One aim of the BELANA trial (Burdens and Expenses of Living as Adult with Nutrition based Allergy or Intolerance) was to study the use of, and satisfaction with, Internet information sources by those strongly affected by food allergies.

## Methods

314 study participants (≥ 18) with self-reported food allergies or intolerance were recruited in 2009 by the German allergy and asthma association (DAAB). 247 completed the BELANA questionnaire four times within four-month intervals. For further evaluation, 117 were considered as food allergic patients, having at least a positive blood and/or skin prick test as well as clinical symptoms. These patients where included in the evaluation of the question, if they had used Internet resources to inform themselves about food product ingredients during the preceding four months. They also had to state their satisfaction with the online information provided.

## Results

46% (average of all single surveys, SD 7.2) of the food allergy sufferers used the Internet to inform themselves about ingredients in food products. While the majority (60%, SD 3.7) tended to go to the food producers' websites, the most of them (36.5% SD 4.6) were not satisfied with the information retrieved; 27.7% (SD 3) were undecided and only 34.5% were pleased (SD 3) with the given information. With 56.7% (SD 6.8), the second most used information resources were forums and chats. Two out of three patients 66.7% (SD 3.4) were satisfied with the information, only 10% (SD 1.5) were unsatisfied, 23% (SD 4.5) were indecisive. Further, 33.9% (SD 4.9) consulted manufacturer-independent product databases, such as WikiFood.eu. The use of these was rated better than online-services of food producers: 50% (SD 5.7) satisfied, 12.4% (SD 1.5) not satisfied and 38.6% (SD 11.4) were indecisive.

## Conclusion

Almost every second particular affected food allergy sufferer uses the Internet to learn about food product ingredients. It turned out that food manufacturers online resources are often not suitable for those concerned. In contrast, the greatest benefit is achieved by using manufacturer-independent product databases as well as chats and forums. However, the provided data quality provided in chats and forums is at least questionable.

## Disclosure of interest

None declared.

